# The Novavax Heterologous Coronavirus Disease 2019 Booster Demonstrates Lower Reactogenicity Than Messenger RNA: A Targeted Review

**DOI:** 10.1093/infdis/jiad519

**Published:** 2023-11-22

**Authors:** Anthony M Marchese, Matthew Rousculp, John Macbeth, Hadi Beyhaghi, Bruce T Seet, Seth Toback

**Affiliations:** Department of Medical Affairs, Novavax, Inc, Gaithersburg, Maryland; Department of Medical Affairs, Novavax, Inc, Gaithersburg, Maryland; Department of Medical Affairs, Novavax, Inc, Gaithersburg, Maryland; Department of Medical Affairs, Novavax, Inc, Gaithersburg, Maryland; Department of Medical Affairs, Novavax, Inc, Gaithersburg, Maryland; Department of Molecular Genetics, University of Toronto, Canada; Department of Medical Affairs, Novavax, Inc, Gaithersburg, Maryland

**Keywords:** COVID-19, booster, reactogenicity, NVX-CoV2373, mRNA vaccines

## Abstract

Coronavirus disease 2019 (COVID-19) continues to be a global health concern, and booster doses are necessary for maintaining vaccine-mediated protection, limiting the spread of severe acute respiratory syndrome coronavirus 2. Despite multiple COVID-19 vaccine options, global booster uptake remains low. Reactogenicity, the occurrence of adverse local/systemic side effects, plays a crucial role in vaccine uptake and acceptance, particularly for booster doses. We conducted a targeted review of the reactogenicity of authorized/approved messenger RNA (mRNA) and protein-based vaccines demonstrated by clinical trials and real-world evidence. It was found that mRNA-based boosters show a higher incidence and an increased severity of reactogenicity compared with the Novavax protein-based COVID-19 vaccine (NVX-CoV2373). In a recent study from the National Institute of Allergy and Infectious Diseases, the incidence of pain/tenderness, swelling, erythema, fatigue/malaise, headache, muscle pain, or fever was higher in individuals boosted with BNT162b2 (0.4% to 41.6% absolute increase) or mRNA-1273 (5.5% to 55.0% absolute increase) compared with NVX-CoV2373. Evidence suggests that NVX-CoV2373, when utilized as a heterologous booster, demonstrates less reactogenicity compared with mRNA vaccines, which, if communicated to hesitant individuals, may strengthen booster uptake rates worldwide.

***Clinical Trials Registration*** NCT04889209.

## Graphical Abstract

**Graphical Abstract:**
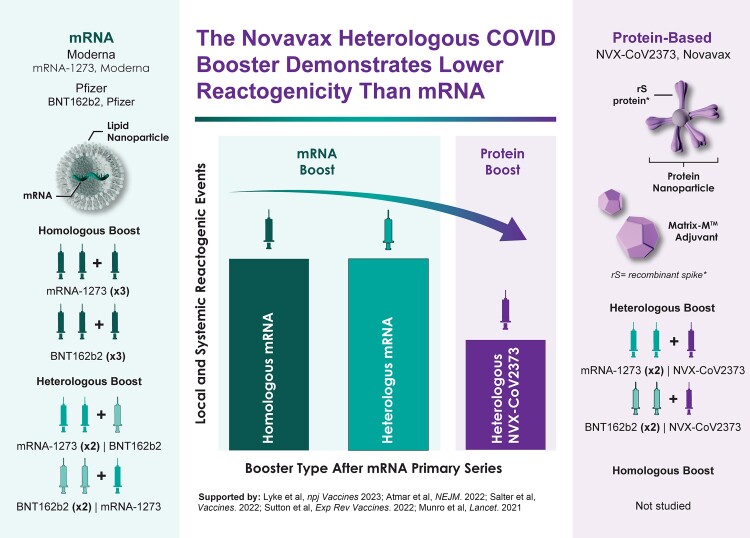

## Teaser

Clinical and real-world evidence studies suggest that mRNA-based boosters induce increased reactogenicity compared with the Novavax protein-based COVID-19 vaccine, NVX-CoV2373. Pain/tenderness, swelling, fatigue, headache, muscle pain, and fever are less common for NVX-CoV2373 versus mRNA boosters.

## THE IMPACT OF REACTOGENICITY ON BOOSTER UPTAKE

As of mid-2023, a high percentage of the global population has received primary series vaccinations for coronavirus disease 2019 (COVID-19), though significantly fewer people have received at least one booster dose. Vaccine reactogenicity, defined as local and systemic reactions, has been identified as one of the leading drivers of vaccine and booster hesitancy [1–4]. Survey data from a nationally representative sample of US adults presented at the September 2023 Advisory Committee on Immunization Practices meeting indicated that adults 18–49 years of age were less likely than adults 50–64 years of age to get an updated COVID-19 vaccine, though both groups were less likely to be vaccinated than adults aged ≥65 years [5]. The higher prevalence of hesitancy among younger adults enhances the relevance of this information, which could impact uptake in the population. Prior experience with vaccine-associated side effects may have contributed to the observed differences, as reactogenicity is often higher in adults <65 years of age compared to adults ≥65 years of age. In other studies, the side effects associated with messenger RNA (mRNA) vaccinations have been shown to disrupt work activities, lead to workplace absenteeism among healthcare workers [2, 6, 7], and reduce future willingness to receive booster vaccinations [2, 3]. Surveys have found that the acceptance rate of various hypothetical vaccines was dependent on the incidence and severity of side effects, and that participants would prefer vaccines with less reactogenicity [1, 8], regardless of efficacy differences [8]. A Health Canal survey found that adults aged ≥65 years were hesitant to receive the bivalent mRNA booster shot and reported that the top reasons for not receiving the vaccine were not knowing if the newly formulated vaccine was safe (40.73%) and concern over potential side effects (31.05%). At the time of the survey, only 42.4% of older adults, a demographic of high-risk for COVID-19, had opted to receive an updated vaccine dose [9]. Vaccine hesitancy is often linked to a fear of adverse reactions, and this may be exacerbated as the COVID-19 pandemic transitions to an endemic state. As the perceived risk of COVID-19 decreases, individuals may become more sensitive to vaccine-associated adverse reactions. With the increasing number of adult vaccines, including those that help protect against influenza and respiratory syncytial virus, simultaneous administration with COVID-19 vaccines may improve uptake, facilitate catch-up, and reduce the number of visits, but any increase will likely rely on the communication of acceptable adverse reaction rates [10, 11].

## EVALUATION OF PLATFORM-SPECIFIC COVID-19 VACCINE REACTOGENICITY

The incidence and severity of reactogenicity following primary series vaccination is well understood from the outcomes of large clinical trials [12–18]. A recent systematic review and meta-analysis compared the local and systemic reactogenicity of primary series mRNA (including BNT162b2 [Pfizer Inc, New York, New York] and mRNA-1273 [Moderna Inc, Cambridge, Massachusetts]), adenovirus vector (Ad26.COV2.S [Janssen Pharmaceuticals, Beerse, Belgium], ChAdOx1 [AstraZeneca plc, Cambridge, United Kingdom], and Ad5-nCov [CanSino Biological, Tianjin, China]), protein-based (NVX-CoV2373 [Novavax Inc, Gaithersburg, Maryland], MVC-COV1901 [Medigen, Taipei, Taiwan], and SCB-2019 [Clover Biopharmaceuticals, Shanghai, China]), and inactivated (BBV152 [Bahrat Biotech, Hyderabad, India], BIBP [Sinopharm, Shanghai, China], and CoronaVac [Sinovac, Beijing, China]) vaccines [19]. The study concluded that vaccine platform type influences the degree of adverse events and that mRNA vaccines were the most reactogenic when compared with viral vector, protein-based, and inactivated vaccines [19]. The lack of standardization of COVID-19 vaccine trial designs such as vaccination and data collection schedules, variable data recording methods, inclusion of different symptom types, and the use of various reactogenicity and severity definitions and grading schemes makes these comparisons challenging [19].

Despite the widespread utilization of different vaccine technologies for primary series vaccinations, recommendations for future booster vaccinations will likely focus on mRNA and protein-based platforms. Viral vector–based vaccines, such as Ad26.COV2.S, are not ideal for boosting due to development of vector-specific immunity [20–22]. By comparison, mRNA and protein-based technologies offer higher vaccine efficacy, a demonstrated safety profile, and in the case of protein vaccines, more manageable storage/handling characteristics. Dependent on regulatory guidelines, many prospective vaccine recipients can receive a booster dose with a different vaccine type than that of their primary series vaccine (a heterologous booster) [23–25].

## METHODS

A targeted literature review was conducted to characterize the reactogenicity of mRNA and NVX-CoV2373 COVID-19 booster vaccination following any mRNA primary series regimen. Using the ProQuest Dialog platform the following databases were searched with no date restraints: BIOSIS Previews, Embase, Embase Preprints, Medline, and publicly available content. This targeted literature search included articles published up to July 2023. Title and abstract terms were searched for “NVX-CoV2373, or Nuvaxovid, or Novavax” and “mRNA, or mRNA-1273, or BNT162, or Moderna, or Pfizer.” Targeted publications included clinical trials or real-world evidence studies that assessed the reactogenicity of NVX-CoV2373 and/or an mRNA vaccine used as boosters following any mRNA primary series vaccination regimen. Only studies that reported booster dose reactogenicity (local and/or systemic adverse events) were included in this review. Similarly, studies that did not include participants with mRNA primary series vaccination regimen, those that solely focused on immunocompromised populations, and studies on nonhuman subjects were excluded from this review.

## RESULTS

A National Institute of Allergy and Infectious Diseases (NIAID) study titled “A Phase 1/2 Study of Delayed Heterologous SARS-CoV-2 Vaccine Dosing (Boost) After Receipt of Emergency Use Authorization (EUA) Vaccines” (NCT04889209) has generated two publications (Table 1) [26, 27]. Taken together, they allow for a comparison of booster dose reactogenicity following different vaccine regimens. In brief, this study is an ongoing, open-label, nonrandomized, adaptive-design clinical trial in adults ≥18 years of age within the United States. The adaptive design permits the addition of new study arms and an increase in sample size as vaccines are awarded Emergency Use Authorization by the US Food and Drug Administration (FDA) and/or as updated variant-adapted vaccines become available. The study aims to assess the safety, reactogenicity, and immunogenicity of a delayed (≥12 weeks) homologous and heterologous vaccine boost following primary series administration of authorized/approved COVID-19 vaccines. Primary series vaccine regimens include two vaccinations of mRNA-1273 (Moderna, 100 µg dose), two vaccinations of BNT162b2 (Pfizer, 30 µg), or one or two vaccination(s) of Ad26.COV2.S (Janssen, 5 × 10^10^ viral particles). The mRNA-1273 vaccine booster dose studied was higher (100 µg) than the currently approved dose, which is 50 µg. The study has currently produced two publications: publication 1 includes individuals who were boosted with mRNA-1273, BNT162b2, or Ad26.COV2.S COVID-19 vaccines, thus providing nine different combinations of primary vaccination and booster [26]; publication 2 includes participants boosted with NVX-CoV2373 (5 µg recombinant spike [rS] protein + 50 µg Matrix-M adjuvant) (Table 1). Both publications had populations with relatively small sample sizes, and those boosted with NVX-CoV2373 were slightly younger than those boosted with mRNA vaccines (Table 1). Data regarding local and systemic reactogenicity were recorded using a memory aid survey and documented on a data collection form for 7 days postvaccination. For reactogenicity severity measurement, each given sign or symptom for a participant's reactogenicity event was counted once under the maximum severity for all postadministration assessments. Participants were also assessed for delayed-onset local reactions through 14 days after each vaccination.

**Table 1.** Demographic Information

Characteristic	NCT04889209 (ClinicalTrials.gov)					
	Atmar et al [26]				Lyke et al [27]	
Primary series (×2)	mRNA-1273	BNT162b2	mRNA-1273	BNT162b2	mRNA-1273	BNT162b2
Booster	mRNA-1273	mRNA-1273	BNT162b2	BNT162b2	NVX-CoV2373	NVX-CoV2373
No. of participants	51	50	51	50	16	31
Age, y, mean (SD)	53 (16)	55 (17)	54 (17)	50 (18)	48.4 (15)	43.2 (12)

Vaccine regimen, number of participants, and age of study participants who received a 2-dose primary series from mRNA-1273 or BNT162b2 followed by a booster dose of mRNA-1273, BNT162b2, or NVX-CoV2373.

Abbreviations: BNT, BioNTech; NVX, Novavax; mRNA, messenger RNA; SD, standard deviation.

**Figure 1.**
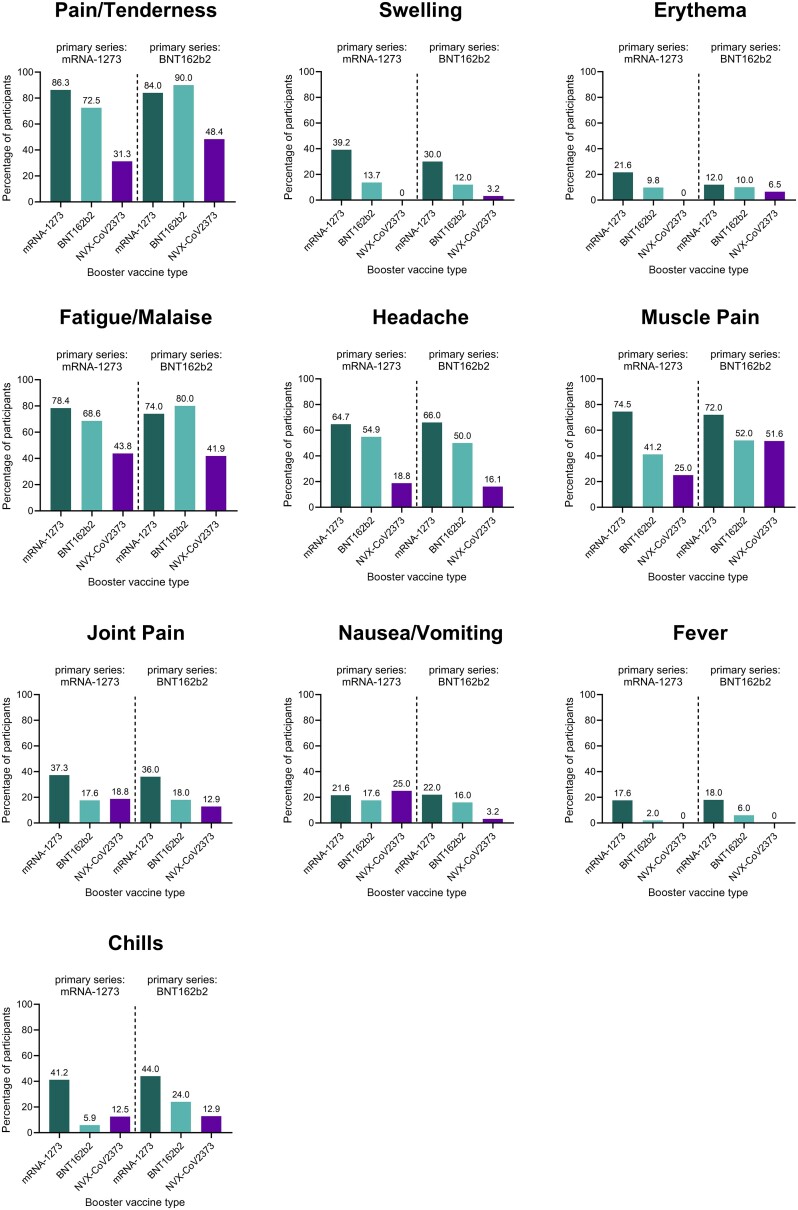
Summary of postvaccination reactogenic events from the National Institute of Allergy and Infectious Diseases study NCT04889209. All participants received a 2-dose primary series vaccination with mRNA-1273 (Moderna) or BNT162b2 (Pfizer), followed by a single booster dose with mRNA-1273 (dark green), BNT162b2 (teal), or NVX-CoV2373 (Novavax Inc, purple). The percentage of participants experiencing any reactogenic event within 7 days postvaccination is shown for the local symptoms of pain/tenderness, swelling, and erythema, and the systemic events of fatigue/malaise, headache, muscle pain, joint pain, nausea/vomiting, fever, and chills. For 3 homologous doses of mRNA-1273, n = 51; mRNA-1273 (×2)/BNT162b2, n = 51; mRNA-1273 (×2)/NVX-CoV2373, n = 16; 3 homologous doses of BNT162b2, n = 50; BNT162b2(×2)/mRNA-1273, n = 50; BNT162b2 (×2)/NVX-CoV2373, n = 31. Except for pain/tenderness for BNT162b2 (×2)/mRNA-1273, n = 49 (one data point reported as missing). Sources: Atmar et al [26] and Lyke et al [27]. Abbreviations: ×2, 2-dose primary series; BNT, BioNTech; mRNA, messenger RNA; NVX, Novavax.

For the purpose of this review, data from participants from publication 1 [26] and publication 2 [27] receiving mRNA (BNT162b2 or mRNA-1273) primary series, followed by mRNA (BNT162b2 or mRNA-1273) or protein-based (NVX-CoV2373) boosters, were summarized as a percentage of participants reporting any symptom and plotted together (Figure 1). These data demonstrate a general pattern of booster-mediated reactogenic event incidence: mRNA-1273 > BNT162b2 > NVX-CoV2373, though the study was not designed to compare between groups. No discernable difference among homologous or heterologous mRNA regimens was clear, as any combination that included mRNA-1273 showed the highest frequency of reactogenicity, except for pain/tenderness (90%) and fatigue/malaise (80%) after 3 homologous doses of BNT162b2. Injection site pain/tenderness was the most common local reaction, with the lowest frequency following mRNA-1273 (×2)/NVX-CoV2373 (31.3%) and BNT162b2 (×2)/NVX-CoV2373 (48.4%) boosters, and the highest frequency following 3 homologous doses of BNT162b2 (90.0%) and 3 homologous doses of mRNA-1273 (86.3%). Fatigue/malaise was the most common systemic reaction, with the lowest frequency following BNT162b2 (×2)/NVX-CoV2373 (41.9%) and mRNA-1273 (×2)/NVX-CoV2373 (43.8%), and the highest frequency following mRNA-1273 (×2)/BNT162b2 (80.0%) and 3 homologous doses of mRNA-1273 (78.4%). Overall, systemic reactions were highest following the mRNA-1273 booster, with two exceptions—fatigue/malaise was most frequent following 3 homologous doses of BNT162b2 (80.0%), and nausea/vomiting was most frequent following the heterologous mRNA-1273 (×2)/NVX-CoV2373 (25.0%). Compared with the mRNA boosters, the NVX-CoV2373 vaccine demonstrated the lowest frequency of pain/tenderness, swelling, erythema, fatigue/malaise, headache, and fever, and displayed similar incidences of muscle pain, joint pain, and nausea/vomiting to the BNT162b2 booster. Detailed severity data included in the primary publications indicated most events being mild or moderate [26, 27]. The NVX-CoV2373 booster was associated with lower or similar severity of local and systemic symptoms compared with mRNA boosters, though the small sample sizes and infrequent occurrence of severe events limit interpretation of the results [26, 27]. The severity of local reactogenicity was generally mild or moderate, as the overall incidence of severe local reactions was low. Pain/tenderness of moderate severity was most frequent following an mRNA-1273 booster (28–30%) as opposed to BNT162b2 (6–20%) and NVX-CoV2373 (6–7%). Similarly, the mRNA-1273 booster had the most frequent moderate-severity swelling (6%), compared with BNT162b2 (0–2%) and NVX-CoV2373 (0–0.03%). No boosters were associated with moderate or severe erythema, except for 1 moderate case after homologous BNT162b2 boosting (2%). Overall, the NVX-CoV2373 booster dose had the lowest severity of local reactogenicity reported [26, 27]. The severity of systemic reactogenicity was predominantly mild or moderate with few severe events. Moderate-severity fatigue, headache, and muscle pain were the most common symptoms and were higher following mRNA-1273 (35–40%, 16–22%, and 20–37%, respectively) and BNT162b2 (20–38%, 8–14%, and 8–18%, respectively) boosters compared to NVX-CoV2373 (13–19%, 0–3%, and 0–7%, respectively) [26, 27]. Similar to local reactogenicity severity results, the NVX-CoV2373 booster had the lowest severity of systemic reactogenicity.

In agreement with NIAID's findings, results from the Evaluating COVID-19 Vaccination Boosters (COV-BOOST) study (ISRCTN73765130) demonstrated parallel patterns of vaccine-induced reactogenicity among the vaccines studied. COV-BOOST is an ongoing, multicenter, randomized, phase 2 trial that aims to assess the safety, reactogenicity, and immunogenicity (at least 70–84 days post–second dose) of homologous and heterologous vaccine boosters following primary series use of BNT162b2 (30 µg) and ChAdOx1-S (5 × 10^10^ viral particles) COVID-19 vaccines in adults aged 30 years and older. The reactogenicity results of COV-BOOST echoed the findings of the NIAID study, showing that the NVX-CoV2373 (5 µg rS + 50 µg Matrix-M) heterologous booster had lower incidence and severity of local and systemic adverse events compared with mRNA-1273 (100 µg) and BNT162b2 (30 µg) boosters [28]. The studied dose of the mRNA-1273 booster (100 µg) was higher than the authorized 50-µg dose. Intriguingly, the studied 30-µg BNT162b2 booster demonstrated a similar profile to that of the 100 µg mRNA-1273, suggesting that mRNA platforms might not display a linear dose-dependent relationship in reactogenicity when administered as a booster. Despite this, the trend of mRNA-1273 showing greater reactogenicity than BNT162b2 has been supported in other studies and surveys [19, 29], though more robust real-world evidence is needed. Additionally, an early pandemic study (Comparing COVID-19 Vaccine Schedule Combinations [Com-COV2]) investigating heterologous primary series regimens similarly found that combinations including mRNA-1273 (100 µg) elicited higher reactogenicity than those including BNT162b2 (30 µg) and that combinations with either mRNA option were generally more reactogenic than those that included NVX-CoV2373 [30].

Due to the timing of NVX-CoV2373 authorization, minimal real-world evidence is currently available. A recent prospective, observational study of US and Canadian working adults demonstrated that reactogenic events captured via patient symptom diaries were approximately 18% lower in NVX-CoV2373–boosted individuals compared with those boosted with mRNA (in this analysis, mRNA-1273 [50 µg] and BNT162b2 [30 µg] were grouped together) [31]. In this study, mRNA boosters elicited a higher percentage of reported symptoms compared to NVX-CoV2373 for injection site tenderness (+25.4% absolute increase vs NVX-CoV2373), injection site pain (+30.4%), muscle pain (+30.8%), swelling (+22.4%), redness (+13%), fatigue (+25.7%), malaise (+24.9%), headache (+18.3%), joint pain (+17.5%), fever (+12%), and nausea/vomiting (+3.5%). Separately, an ongoing, publicly available survey from the Japanese Ministry of Health, Labor and Welfare studied real-world, self-reported reactogenicity of mRNA-1273, BNT162b2, and NVX-CoV2373 boosters and similarly found a reactogenicity trend of mRNA-1273 > BNT162b2 > NVX-CoV2373; among booster participants, dependent on primary series, 85%–88% of NVX-CoV2373, 28%–67% of BNT162b2, and 14%–41% of mRNA-1273 recipients reported feeling better compared to their second primary series dose [32]. A prospective cohort study of Australian community pharmacy vaccinations found that respondents receiving mRNA-1273 (54.7%) or BNT162b2 (41.6%) boosters were more likely to report any adverse event (local reaction, systemic aches, fatigue, fever, chills, gastrointestinal, rash, fainting, seizure, or other) following immunization than those receiving NVX-CoV-2373 (28.7%) [29]. In an analysis of adverse events reported to the Korean Disease Control and Prevention Agency (South Korea) between February 26, 2021 and July 31, 2022, BNT162b2, mRNA-1273, and NVX-CoV2373 were compared. This analysis found that headache, muscle pain, and vertigo/dizziness were the most common adverse reactions. Among the vaccines of interest, the rates of suspected adverse reactions by type of COVID-19 vaccine were highest in mRNA-1273 (541 per 100 000 cases), followed by BNT162b2 (452 per 100 000 cases), and were the lowest after NVX-CoV2373 (142 per 100 000 cases) [33].

## DISCUSSION

Though NVX-CoV2373 is a protein-based vaccine, the results of these studies should not be directly applied to other protein-based options. Due to the inherently low immunogenicity of recombinant proteins, adjuvants are typically used to enhance the vaccine-mediated immune response of protein-based platforms [34, 35]. The currently authorized and investigational protein-based COVID-19 vaccines each utilize different adjuvants that may lead to unique reactogenicity profiles. NVX-CoV2373 includes a saponin-based adjuvant known as Matrix-M, while other vaccines utilize an oil-in-water emulsion such as SQBA (HIPRA), aluminum hydroxide gel and CpG1018 (Clover Biopharmaceuticals, Biological E. Limited), or squalene-based AS03 (SK Bioscience Co, GSK plc, Sanofi) [36]. Uniquely, Matrix-M has been found to have a time-restricted occurrence at the injection site and to rapidly distribute to the draining lymph nodes, which is thought to support its favorable reactogenicity profile [37]. The variation among the protein-based vaccines creates the need for additional studies to compare booster dose reactogenicity among different protein-based COVID-19 platforms to understand the impacts of the adjuvants, protein, and dose level.

The overview discussed here is limited by the current paucity of studies, as few rigorous comparative studies are available. In these studies, no homologous booster regimens of NVX-CoV2373 were investigated due to the timing of vaccine availability and logistics. Additionally, we chose to exclude discussion of viral vector and other vaccine platforms to develop a more focused assessment of mRNA and protein-based boosters. An additional assessment that includes investigation of viral vector vaccines used as primary series vaccinations may be warranted to understand the potential combinations of vaccines and their outcomes more completely. A comparative analysis designed to test differences among vaccine options is warranted. Future research that investigates the duration of symptoms and the incidence of rare events that have been infrequently observed, such as severe local and systemic symptoms, would be informative. This targeted review was unable to assess the observation that some vaccines have shown a correlation between increased neutralizing antibodies and increased reactogenicity [38, 39], and the clinical significance (i.e., impact on vaccine effectiveness) of this observation remains unknown. Future real-world studies investigating the impacts of reactogenicity on productivity and school/workplace absenteeism would offer more detailed insights into vaccine-specific side effects and their societal consequences.

## CONCLUSIONS

The results of different trials and real-world evidence have shown that the NVX-CoV2373 heterologous booster was associated with reduced incidence and severity of local and systemic adverse events compared with mRNA-1273 and BNT162b2. The reactogenicity of COVID-19 vaccines may become a focal point as the world transitions from a pandemic to an endemic state. Though the reviewed data included vaccines with compositions based on the original Wuhan spike protein, in the absence of changes to dose concentrations or vaccine components (i.e., mRNA vaccine lipid nanoparticles, or protein-based vaccine adjuvant), it is unlikely that the updated variant-based formulations will have a significant impact on the observed reactogenicity trends. These findings provide practical insights for healthcare providers and the public as they weigh the benefits and risks of additional COVID-19 vaccinations.

## Notes

***Author contributions.*** A. M. M. and S. T. developed the concept for the review article. A. M. M. wrote the initial draft and generated the table and figure. M. R., H. B., J. M., and B. T. S. provided scientific expertise and edited the article. All authors approved of the final draft.

***Acknowledgments.*** Research, medical, and scientific support was provided by Sharon Glass and Elizabeth A. Kitchens, PhD, who are employees of Novavax, Inc. The graphical abstract was created by Emily Adams, formerly of Novavax, Inc., and currently a freelance medical illustrator at emadamsinc.com.

***Financial support.*** Medical writing and editing support for the development of this viewpoint, under the direction of the authors, was provided by Rebecca Harris, PhD, and Amanda Cox, PhD, of Ashfield MedComms (New York, New York), an Inizio company, and was funded by Novavax, Inc.

## References

[1] Al-Obaydi S , Hennrikus E, Mohammad N, Lehman EB, Thakur A, Al-Shaikhly T. Hesitancy and reactogenicity to mRNA-based COVID-19 vaccines—early experience with vaccine rollout in a multi-site healthcare system. PLoS One 2022; 17:e0272691. 35930586 10.1371/journal.pone.0272691 PMC9355214

[2] Chrissian AA , Oyoyo UE, Patel P, et al Impact of COVID-19 vaccine–associated side effects on health care worker absenteeism and future booster vaccination. Vaccine 2022; 40:3174–81. 35465979 10.1016/j.vaccine.2022.04.046 PMC9013647

[3] Long S , Wu J, Wang S, et al Changes of factors associated with vaccine hesitancy in Chinese residents: a qualitative study. Front Public Health 2022; 10:929407. 36203693 10.3389/fpubh.2022.929407 PMC9530596

[4] Rief W . Fear of adverse effects and COVID-19 vaccine hesitancy: recommendations of the treatment expectation expert group. JAMA Health Forum 2021; 2:e210804. 36218819 10.1001/jamahealthforum.2021.0804

[5] Wallace M , Centers for Disease Control and Prevention. Evidence to recommendations framework: 2023–2024 (monovalent, XBB containing) COVID-19 vaccine. Advisory Committee on Immunization Practices (ACIP). 2023. https://www.cdc.gov/vaccines/acip/meetings/downloads/slides-2023-09-12/11-covid-wallace-508.pdf. Accessed 11 November 2023.

[6] Breeher LE , Wolf ME, Geyer H, et al Work absence following COVID-19 vaccination in a cohort of healthcare personnel. J Occup Environ Med 2022; 64:6–9. 34982070 10.1097/JOM.0000000000002376 PMC8715930

[7] Cohen DA , Greenberg P, Formanowski B, Parikh PD. Are COVID-19 mRNA vaccine side effects severe enough to cause missed work? Cross-sectional study of health care-associated workers. Medicine (Baltimore) 2022; 101:e28839. 35363178 10.1097/MD.0000000000028839 PMC9282130

[8] Rosiello DF , Anwar S, Yufika A, et al Acceptance of COVID-19 vaccination at different hypothetical efficacy and safety levels in ten countries in Asia, Africa, and South America. Narra J 2021; 1:e55. 38450212 10.52225/narra.v1i3.55 PMC10914086

[9] Costa K . Older adults’ intentions and attitudes toward the updated bivalent COVID-19 booster 2023. 2023. https://www.healthcanal.com/health/the-bivalent-covid-19-booster-survey. Accessed 7 June 2023.

[10] World Health Organization . Mitigating the impact of COVID-19 on control of vaccine-preventable diseases: a health risk management approach focused on catch-up vaccination. 2020. https://www.who.int/europe/publications/i/item/WHO-EURO-2020-1086-40832-55187. Accessed 11 November 2023.

[11] Centers for Disease Control and Prevention What to know about getting flu COVID-19 and RSV vaccines at the same time. 2023. https://www.cdc.gov/respiratoryviruses/whats-new/getting-vaccines-at-same-time.html#print. Accessed 11 November 2023.

[12] Áñez G , Dunkle LM, Gay CL, et al Safety, immunogenicity, and efficacy of the NVX-CoV2373 COVID-19 vaccine in adolescents: a randomized clinical trial. JAMA Netw Open 2023; 6:e239135. 37099299 10.1001/jamanetworkopen.2023.9135 PMC10536880

[13] Dunkle LM , Kotloff KL, Gay CL, et al Efficacy and safety of NVX-CoV2373 in adults in the United States and Mexico. N Engl J Med 2021; 386:531–43. 34910859 10.1056/NEJMoa2116185 PMC8693692

[14] El Sahly HM , Baden LR, Essink B, et al Efficacy of the mRNA-1273 SARS-CoV-2 vaccine at completion of blinded phase. N Engl J Med 2021; 385:1774–85. 34551225 10.1056/NEJMoa2113017 PMC8482810

[15] Falsey AR , Sobieszczyk ME, Hirsch I, et al Phase 3 safety and efficacy of AZD1222 (ChAdOx1 nCoV-19) Covid-19 vaccine. N Engl J Med 2021; 385:2348–60. 34587382 10.1056/NEJMoa2105290 PMC8522798

[16] Heath PT , Galiza EP, Baxter DN, et al Safety and efficacy of NVX-CoV2373 Covid-19 vaccine. N Engl J Med 2021; 385:1172–83. 34192426 10.1056/NEJMoa2107659 PMC8262625

[17] Sadoff J , Gray G, Vandebosch A, et al Safety and efficacy of single-dose Ad26.COV2.S vaccine against Covid-19. N Engl J Med 2021; 384:2187–201. 33882225 10.1056/NEJMoa2101544 PMC8220996

[18] Thomas SJ , Perez JL, Lockhart SP, et al Efficacy and safety of the BNT162b2 mRNA COVID-19 vaccine in participants with a history of cancer: subgroup analysis of a global phase 3 randomized clinical trial. Vaccine 2022; 40:1483–92. 35131133 10.1016/j.vaccine.2021.12.046 PMC8702495

[19] Sutton N , San Francisco Ramos A, Beales E, et al Comparing reactogenicity of COVID-19 vaccines: a systematic review and meta-analysis. Expert Rev Vaccines 2022; 21:1301–18. 35796029 10.1080/14760584.2022.2098719

[20] Deng S , Liang H, Chen P, et al Viral vector vaccine development and application during the COVID-19 pandemic. Microorganisms 2022; 10:1450. 35889169 10.3390/microorganisms10071450 PMC9317404

[21] Kunal S , Sakthivel P, Gupta N, Ish P. Mix and match COVID-19 vaccines: potential benefit and perspective from India. Postgrad Med J 2022; 98:e99–e101. 37066538 10.1136/postgradmedj-2021-140648

[22] Verdecia M , Kokai-Kun JF, Kibbey M, Acharya S, Venema J, Atouf F. COVID-19 vaccine platforms: delivering on a promise? Hum Vaccin Immunother 2021; 17:2873–93. 34033528 10.1080/21645515.2021.1911204 PMC8381795

[23] Centers for Disease Control and Prevention . Summary document for interim clinical considerations for use of COVID-19 vaccines currently authorized in the United States. 2022. https://stacks.cdc.gov/view/cdc/123540. Accessed 5 January 2023.

[24] European Medicines Agency . Nuvaxovid: EPAR—product information. 2023. https://www.ema.europa.eu/en/documents/product-information/nuvaxovid-epar-product-information_en.pdf. Accessed 7 June 2023.

[25] Public Health Agency of Canada . Summary of NACI statement of January 20, 2023: guidance on COVID-19 vaccine booster doses: initial considerations for 2023. 2023. https://www.canada.ca/en/public-health/services/immunization/national-advisory-committee-on-immunization-naci/guidance-covid-19-vaccine-booster-doses-initial-considerations-2023/summary-january-20-2023.html. Accessed 7 June 2023.

[26] Atmar RL , Lyke KE, Deming ME, et al Homologous and heterologous Covid-19 booster vaccinations. N Engl J Med 2022; 386:1046–57. 35081293 10.1056/NEJMoa2116414 PMC8820244

[27] Lyke KE , Atmar RL, Dominguez Islas C, et al Immunogenicity of NVX-CoV2373 heterologous boost against SARS-CoV-2 variants. NPJ Vaccines 2023; 8:98. 37433788 10.1038/s41541-023-00693-z PMC10336079

[28] Munro APS , Janani L, Cornelius V, et al Safety and immunogenicity of seven COVID-19 vaccines as a third dose (booster) following two doses of ChAdOx1 nCov-19 or BNT162b2 in the UK (COV-BOOST): a blinded, multicentre, randomised, controlled, phase 2 trial. Lancet 2021; 398:2258–76. 34863358 10.1016/S0140-6736(21)02717-3 PMC8639161

[29] Salter SM , Li D, Trentino K, et al Safety of four COVID-19 vaccines across primary doses 1, 2, 3 and booster: a prospective cohort study of Australian community pharmacy vaccinations. Vaccines (Basel) 2022; 10:2017. 36560426 10.3390/vaccines10122017 PMC9786585

[30] Stuart ASV , Shaw RH, Liu X, et al Immunogenicity, safety, and reactogenicity of heterologous COVID-19 primary vaccination incorporating mRNA, viral-vector, and protein-adjuvant vaccines in the UK (Com-COV2): a single-blind, randomised, phase 2, non-inferiority trial. Lancet 2022; 399:36–49. 34883053 10.1016/S0140-6736(21)02718-5 PMC8648333

[31] Rousculp M , Ziemiecki R, Marchese AM. Protein vaccine demonstrates less reactogenicity than mRNA—a real world study. medRxiv [Preprint]. Posted online 4 June 2023. doi:10.1101/2023.05.31.23290594

[32] Ministry of Health Labor and Welfare of Japan . FY2020 Ministry of Health, Labor and Welfare commissioned project: interim report on health situation survey after COVID-19 vaccination. 2022. https://www.mhlw.go.jp/content/10601000/000997973.pdf. Accessed 7 June 2023.

[33] Kim SH , Yon DK, Choi YS, et al Vertigo and dizziness after coronavirus disease-2019 vaccination: a nationwide analysis. J Int Adv Otol 2023; 19:228–33. 37272641 10.5152/iao.2023.22937 PMC10331640

[34] Di Pasquale A , Preiss S, Tavares Da Silva F, Garcon N. Vaccine adjuvants: from 1920 to 2015 and beyond. Vaccines (Basel) 2015; 3:320–43. 26343190 10.3390/vaccines3020320 PMC4494348

[35] Reed SG , Orr MT, Fox CB. Key roles of adjuvants in modern vaccines. Nat Med 2013; 19:1597–608. 24309663 10.1038/nm.3409

[36] Marchese AM , Beyhaghi H, Orenstein WA. With established safe and effective use, protein vaccines offer another choice against COVID-19. Vaccine 2022; 40:6567–9. 36210248 10.1016/j.vaccine.2022.09.064 PMC9515329

[37] Carnrot C CB , Palm AKE, Akpinar E, et al Biodistribution of the saponin-based adjuvant Matrix-M following intramuscular injection in mice. Front Drug Deliv 2023; 3:1279710. 10.3389/fddev.2023.1279710 PMC12363263 40838051

[38] Dutcher EG , Epel ES, Mason AE, et al The more symptoms the better? Covid-19 vaccine side effects and long-term neutralizing antibody response. medRxiv [Preprint]. Posted online 3 November 2023. doi:10.1101/2023.09.26.23296186

[39] Hermann EA , Lee B, Balte PP, et al Association of symptoms after COVID-19 vaccination with anti-SARS-CoV-2 antibody response in the Framingham Heart Study. JAMA Netw Open 2022; 5:e2237908. 36269359 10.1001/jamanetworkopen.2022.37908 PMC9587476

